# Emergence of hypervirulent and carbapenem-resistant *Klebsiella pneumoniae* ST37: genomic and phenotypic characterization of virulence and resistance

**DOI:** 10.3389/fmicb.2026.1741536

**Published:** 2026-01-26

**Authors:** Li Shen, Weihua Han, Yu Huang, Shanshan Wang, Haojin Gao, Jinjin Yang, Jiana Fu, Zhixuan Chen, Junhong Shi, Xinru Yuan, Jingyi Yu, Fangyou Yu, Chunyang Wu

**Affiliations:** 1 Department of Clinical Laboratory, Shanghai Pulmonary Hospital, School of Medicine, Tongji University, Shanghai, China; 2 Department of Clinical Laboratory Medicine, The First Affiliated Hospital of Ningbo University, Ningbo, China; 3 Department of Endocrinology, The First Affiliated Hospital of Wenzhou Medical University, Wenzhou, China

**Keywords:** aerobactin, carbapenem-resistant, hypervirulent, *Klebsiella pneumoniae*, siderophore, ST37

## Abstract

The emergence and spread of carbapenem-resistant *Klebsiella pneumoniae* (CRKP) ST37 necessitates urgent reassessment of its pathogenic potential. This study aims to characterize the genomic and phenotypic features of ST37 isolates collected from a tertiary hospital in eastern China, from December 2020 to August 2023. Fifteen CRKP isolates underwent antimicrobial susceptibility testing, whole-genome sequencing (WGS), and phylogenetic analysis for resistance/virulence gene profiling. Phenotypic virulence assays, including quantitative siderophore production, string test, mucoviscosity, capsule quantification, serum resistance, and *Galleria mellonella in vivo* infection model were designed to evaluate the level of pathogenicity of the isolates. All ST37 *Klebsiella pneumoniae* isolates exhibited resistance to carbapenems, with 60.0% (9/15) harboring *bla*_KPC-2_ alongside extensive ESBL genes. Critically, a significant subset (26.7%, 4/15; YF1062/YF1072/YF2197/YF2203; KL25/O5) demonstrated hypervirulent phenotypes, evidenced by elevated siderophore production, aerobactin gene carriage (*iucABCD*/*iutA*), and extreme lethality in *Galleria mellonella*, confirming convergent ST37 CRKP-hvKP emergence. This study confirms the emergence of hypervirulent strains within the carbapenem-resistant ST37 lineage, representing a significant convergent threat necessitating urgent surveillance and novel interventions.

## Introduction

The relentless rise of antimicrobial resistance (AMR) constitutes a global health emergency, with carbapenem-resistant *Enterobacterales* (CRE) posing a paramount threat due to limited therapeutic options and high mortality (Tacconelli et al., 2018). *Klebsiella pneumoniae*, a leading cause of severe healthcare-associated infections, exemplifies this crisis through the global dissemination of carbapenem-resistant *K. pneumoniae* (CRKP) clones (Wang et al., 2022). Resistance, primarily mediated by carbapenemases encoded on mobile genetic elements, effectively dismantles the efficacy of last-resort β-lactam antibiotics (Huynh et al., 2020; Queenan and Bush, 2007). Among these enzymes, *K. pneumoniae* carbapenemase (KPC), particularly the KPC-2 variant, has been a dominant driver of CRKP epidemics worldwide (Shen et al., 2009; Lee et al., 2016; Li et al., 2014). Molecular epidemiology reveals that the CRKP pandemic is fueled by the expansion of specific high-risk sequence types (STs) (Chen et al., 2014). ST11, ST14, ST101, ST147 and ST258 are the main clones of carbapenem-producing *K. pneumoniae* (Lee et al., 2016; Zhu et al., 2024; Hu et al., 2020; Chen et al., 2024a). While ST11 and its single-locus (*tonB*) variant, ST258, have been extensively characterized and are globally prevalent (Yang et al., 2013; Qi et al., 2011; Tóth et al., 2010), ST37 is emerging as a concerning epidemic clone demanding increased attention (Li et al., 2017; Zhu et al., 2016). ST37 CRKP strains demonstrate remarkable persistence and transmission within healthcare ecosystems (Guo et al., 2016; Illiaquer et al., 2012; Xia et al., 2017). Critically, recent reports suggest ST37 is increasingly associated with KPC-2 production, distinguishing it from clones often linked to metallo-β-lactamases (Piccirilli et al., 2021; Zhou et al., 2020; Bedenić et al., 2012). This association with a highly transmissible resistance gene underscores the clone’s epidemiological significance.

Multidrug-resistant (MDR) *K. pneumoniae* strains, including many CRKP clones, were considered less virulent than hypervirulent *K. pneumoniae* (hvKP) lineages. In contrast to MDR strains, hvKP typically cause community-acquired, invasive infections in healthy individuals but often remain antimicrobial-susceptible (Lee et al., 2017). Although hypervirulent and antimicrobial-resistant populations of *K. pneumoniae* generally demonstrate limited overlap, clinical isolates co-harboring hypervirulence and multidrug resistance have been documented (Bialek-Davenet et al., 2014). Critically, the emergence of hv-CRKP and CR-hvKP strains exemplifies the convergence of enhanced virulence and multidrug resistance, potentially evolving into a critical clinical challenge (Cejas et al., 2014; Xu et al., 2019; Zhang R. et al., 2015; Zhang Y. et al., 2015; Jiang et al., 2025). *K. pneumoniae* has developed a range of sophisticated strategies to thrive and evade the host’s immune defenses. Currently, four key virulence factors have been identified as pivotal to its survival: capsule, lipopolysaccharide (LPS), siderophores, and fimbriae (Paczosa and Mecsas, 2016). These factors play a crucial role in enhancing its pathogenicity and enabling successful infection, making *K. pneumoniae* a formidable adversary in the fight against infectious diseases. Virulence in *K. pneumoniae* is multifaceted, but efficient iron acquisition is fundamental for survival and proliferation within the hostile, iron-restricted host environment (Russo et al., 2011). Siderophores are high-affinity iron chelators that play a crucial role as pathogenic factors, enabling organisms to acquire iron from their environment or host during conditions of iron deficiency (Khan et al., 2018; Řezanka et al., 2019). *K. pneumoniae* utilizes multiple siderophore systems, including enterobactin, salmochelin, aerobactin, and yersiniabactin (Kumar et al., 2024). Aerobactin, a major siderophore virulence determinant whose production significantly contributes to enhanced iron acquisition (Paczosa and Mecsas, 2016). Aerobactin-positive isolates are linked to more severe infections and represent a key genetic feature of hvKP (Nassif and Sansonetti, 1986; Russo et al., 2014), making aerobactin a potential target for anti-virulence strategies (Russo et al., 2015). The acquisition of such potent siderophores by traditionally “low-virulence” MDR clones represents a critical evolutionary step toward heightened pathogenicity.

Despite its rising prominence as a CRKP clone, particularly linked to KPC-2, the intrinsic virulence profile of ST37 remains inadequately defined. It is unknown whether this successful resistant clone acquires or expresses potent virulence determinants, such as high-efficiency siderophores, that could potentially offset fitness costs associated with resistance and thereby facilitate more severe disease manifestations. Elucidating the virulence landscape of ST37 CRKP is therefore essential for accurate risk assessment, outcome prediction, and the development of targeted therapeutic and preventive strategies against this expanding threat. Here, we report a disconcerting convergence of resistance and virulence within the ST37 CRKP lineage. Through comprehensive phenotypic and genotypic analysis of a clinical collection, we demonstrate that all studied ST37 isolates are CRKP, with the overwhelming majority harboring the *bla*_KPC-2_ gene. Furthermore, we identify a significant subset exhibiting markedly elevated virulence potential. This hypervirulent phenotype correlates directly with the presence and expression of specific siderophore systems, particularly aerobactin. Our findings reveal that ST37 CRKP is evolving beyond a purely resistant pathogen; it is acquiring the capacity for enhanced pathogenesis, signifying its emergence as a true high-risk clone. This dual threat of pan-resistance and augmented virulence underscores the urgent need for enhanced surveillance, stringent infection control, and accelerated development of novel countermeasures targeting such convergent pathogens.

## Materials and methods

### Bacterial isolates and antimicrobial susceptibility test

Fifteen non-duplicate carbapenem-resistant *K. pneumoniae* (CRKP) isolates were collected from clinical specimens of distinct patients at the First Affiliated Hospital of Ningbo University, from December 2020 to August 2023. The information about the isolates were listed in Table 1. All isolates were confirmed as *K. pneumoniae* by MALDI-TOF MS (BioMerieux, France). The hypervirulent *K. pneumoniae* strain NTUH-K2044 (hvKP, ST23) and classical *K. pneumoniae* strain HS11286 (cKP, ST11) served as positive and negative virulence controls, respectively. The minimum inhibitory concentrations (MICs) of 15 CRKP isolates were determined by the VITEK2 system according to the Clinical and Laboratory Standards Institute (CLSI) guidelines. *E. coli* ATCC 25922 was included as the quality control strain. The interpretive breakpoints were based on CLSI2024-M100-ED34.

**Table 1:** Bacterial strains used in this study.

Isolate	Isolation date (day/mo/yr)	Gender/age (yr)	Specimen	Ward	Underlying disease or condition
YF1062	3/12/2020	M/50	Pleural fluid	ICU	Small intestinal perforation
YF1072	1/1/2021	M/91	Sputum	ICU	Intertrochanteric fracture
YF1073	1/1/2021	F/89	Sputum	Pulmonology	Chronic obstructive pulmonary disease
YF1078	27/1/2021	M/54	Sputum	ICU	Chronic bronchitis
YF1083	18/2/2021	N/A	Stool	ICU	N/A
YF1101	20/5/2021	M/90	Blood	ICU	Acute renal failure, severe pneumonia
YF2155	7/9/2022	M/92	Sputum	Gerontology	Chronic obstructive pulmonary disease
YF2177	4/11/2022	F/64	Urine	Nephrology	Urinary tract infection
YF2180	13/11/2022	M/80	Urine	Neurology	Myelitis
YF2189	9/5/2023	F/65	Urine	Cancer radio-chemotherapy	Bladder cancer
YF2197	28/1/2023	F/90	Sputum	Neurology	Cerebral infarction
YF2203	1/2/2023	F/90	Stool	Neurology	Cerebral infarction
YF2227	12/3/2023	F/80	Urine	Nephrology	Pulmonary edema
YF2228	17/3/2023	M/74	Blood	ICU	Acute diffuse peritonitis
YF2326	27/8/2023	M/60	Puncture fluid	Hepatobiliary and pancreatic surgery	Fever

N/A, not available.

### Whole genome sequencing and bioinformatics analysis

The genomic DNA of 15 *K. pneumoniae* isolates was extracted with a commercial kit (Qiagen, Germany) and WGS was performed using the Illumina NovaSeq 6,000 platform. Multilocus sequence typing (MLST) was identified using MLST^1^ with the *K. pneumoniae* 7-locus MLST scheme. Antibiotic resistance genes, virulence factors, capsular serotype, and plasmid replicon were comprehensively characterized through bioinformatic analyses with ResFinder^2^, Kleborate^3^, and PlasmidFinder^4^ (Zhu et al., 2024). From all the 120,000 genomes of *K. pneumoniae* obtained from NCBI (accessed in July 2025), we screened out 1,236 strains confirmed to be ST37 for subsequent analysis. The phylogenetic relationship of 1,251 genome sequences (15 obtained in this study and 1,236 downloaded from the public NCBI dataset) was constructed using ParSNP, followed by visualization and annotation using the interactive tree of life (iTOL).

### Siderophore production quantification assay

The iron-chelating capacity of bacterial supernatants was evaluated through chrome azurol S (CAS) assay as previously described (Schwyn and Neilands, 1987). Bacterial suspensions (1 μL) from logarithmic-phase cultures were inoculated onto CAS plates. After 24 h incubation at 37 °C, the siderophore production was determined by measuring orange halos.

### String test

The string test was performed to investigate the hypermucoviscosity phenotype of CRKP strains. Following 24 h incubation on blood agar at 37 °C, colonies were tested by touching with a sterile loop and lifting vertically. Strains demonstrating viscous string >5 mm in length were designated as hypermucoviscous.

### Mucoviscosity assay

The mucoviscosity was measured using a modified protocol essentially as described previously (Nucci et al., 2022; Walker et al., 2019). Overnight bacterial cultures were assessed for initial OD_600_ (pre-centrifugation). After centrifugation ( 2000×g , 5 min), 200 μL supernatant was loaded into 96-well plates for secondary OD_600_ (post-centrifugation) measurement. The post-/pre-centrifugation OD_600_ ratio was computed as an indicator of semi-quantitative mucoviscosity.

### Capsular polysaccharide quantification assay

The assessment of CPS production by uronic acid measurement was performed essentially as described (Khadka et al., 2023). A 500 μL of overnight bacterial culture was mixed with 100 μL of 1% Zwittergent 3–12 detergent and incubated at 50 °C for 20 min. The mixture was centrifuged at 13,000×g for 5 min. Subsequently, 300 μL of supernatant was transferred to a new microcentrifuge tube, mixed with 1.2 mL of absolute ethanol, and incubated on ice for 20 min. Following centrifugation at 13,000×g for 5 min, the supernatant was carefully discarded, and the pellet was air-dried. The precipitate was resuspended in 100 μL of ddH_2_O, then mixed with 600 μL of sodium tetraborate solution (12.5 mM in concentrated sulfuric acid). The solution was boiled at 100 °C for 5 min, immediately cooled on ice for 10 min, and mixed with 10 μL of hydroxydiphenyl reagent. After incubation for 5 min at room temperature, OD_520_ was measured to determine uronic acid concentration.

### Serum resistance assay

To assess the ability of CRKP strains to resist the killing of serum, we conducted a serum resistance assay, following established protocols (Zhou et al., 2025). Bacterial suspensions were adjusted to 1 × 10^6^ CFU/mL, and then, 25 μL aliquots were mixed with 75 μL of healthy human serum and incubated with constant shaking (220 rpm) for 2 h at 37 °C. Samples collected at 0 h and 2 h were serially diluted in sterile PBS, plated onto LB agar, and incubated overnight at 37 °C. Viable bacterial counts were determined, and the survival rate was calculated as: (CFU at 2 h/CFU at 0 h) × 100%.

### *In vivo* virulence assays in *Galleria mellonella*

To verify the pathogenicity of CRKP strains, *G. mellonella* infection model were applied. Larvae in experimental groups were injected with 10 μL aliquots containing bacterial suspensions at 1 × 10^6^ or 1 × 10^7^ CFU/mL, while control groups received equivalent volumes of sterile PBS. Each treatment group contained ≥30 larvae distributed across three independent Petri dishes, and kept at 37 °C. Larval survival was monitored daily over a 4 day experimental period.

### Statistical analysis

All experiments were performed with at least three replicates. Data are expressed as the mean ± standard deviation (mean ± SD). Statistical significance was determined using two-tailed Student’s *t*-test for parametric data and log-rank test for survival analyses in GraphPad Prism 9 software. *p* < 0.05 was considered statistically significant throughout the study.

## Results

### Identification of *K. pneumoniae* ST37

In this study, we collected a total of 15 *K. pneumoniae* isolates retrospectively collected between 2020 and 2023. Epidemiological profiling of the bacterial strains collected, primarily isolated from middle-aged and elderly patients, revealed that sputum and urine were the predominant specimen sources, accounting for 33.3% (1/3) and 26.7% (4/15) of the isolates, respectively. This finding carries significant clinical implications as it underscores the increased susceptibility of this patient demographic to both respiratory and genitourinary infections. The prevalence of sputum isolates highlights the critical role of respiratory pathogens, which may be associated with age-related declines in pulmonary immunity or comorbid conditions. Simultaneously, the substantial yield from urine samples is consistent with established epidemiological trends indicating that urinary tract infections are particularly prevalent among older adults, especially within healthcare-associated environments.

### Analysis of the core genome

**Figure 1:**
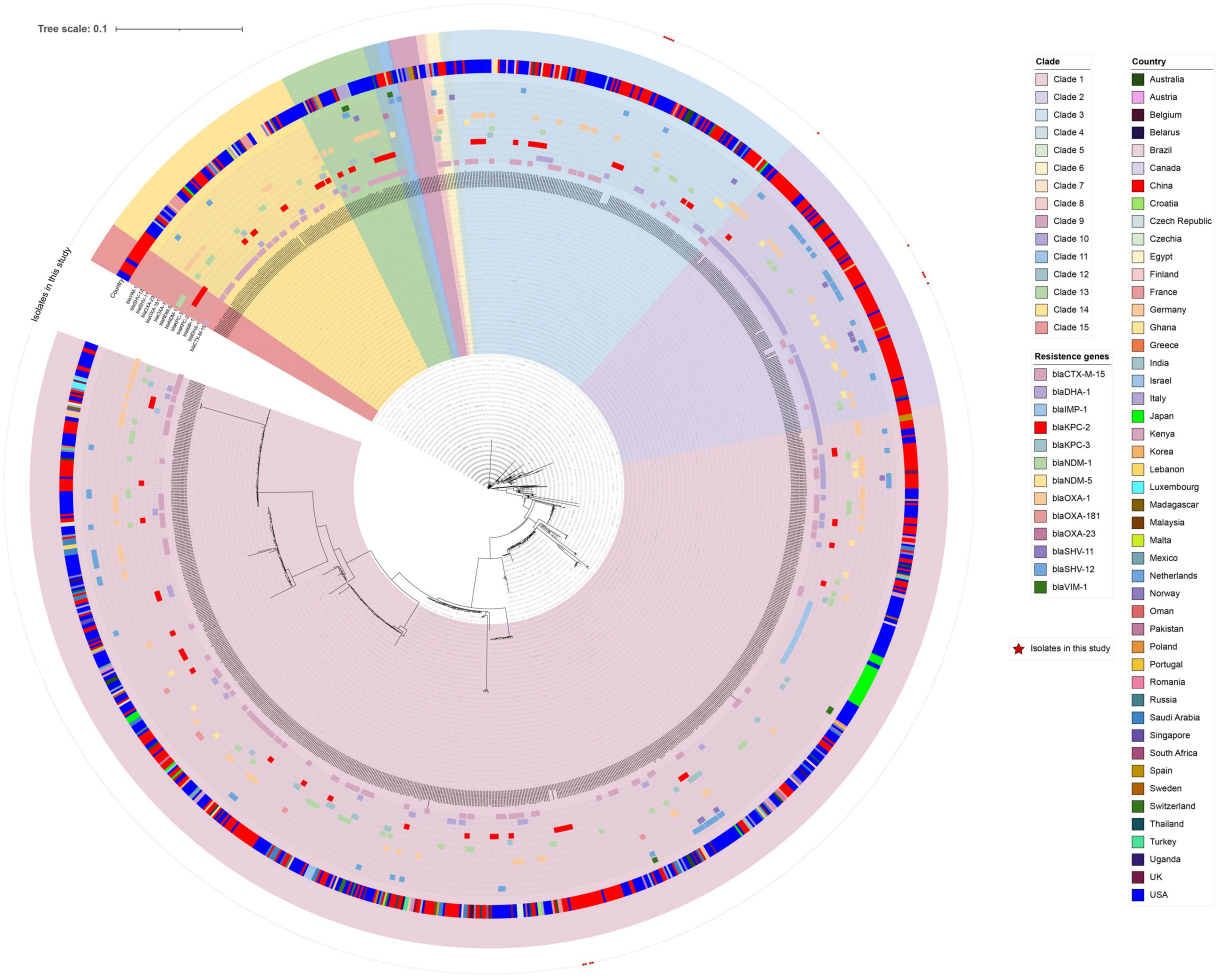
Phylogenetic analysis of *K. pneumoniae* ST37. Colored blocks indicate the presence of specific features in the strains, with the specific features listed in the legend to the right of the phylogenetic tree.

The circular phylogenetic tree provided a compelling illustration of the evolutionary relationships and distribution patterns of bacterial isolates across a variety of clades and countries, highlighting significant resistance genes (Figure 1). In total, we analyzed 1,251 *K. pneumoniae* ST37 isolates, which were meticulously categorized into 15 distinct clades (from Clade 1 to Clade 15). Notably, these clades exhibited diverse geographic distributions, with the majority of isolates concentrated in the United States and China. Our study identified 15 isolates that were specifically grouped within Clade 1, Clade 2, and Clade 3. Through rigorous genomic analysis, we uncovered a wide-ranging resistance gene profile, featuring an array of β-lactamase genes, including the crucial extended-spectrum β-lactamases (ESBLs). Among the global *K. pneumoniae* strains of the ST37 type, a striking 95 strains (accounting for 7.6%) harbor the KPC-2 gene, including 9 strains identified right here in our study. In the whole-genome SNP-based phylogenetic tree, YF2203, YF2197, YF1072, and YF1062 were in clade 1, with a maximum SNP difference of 17 and a minimum SNP difference of 3 compared to the reference strain YF2203. However, YF2326, YF1073, YF2227, YF2189, YF2177, and YF2180 were in clade 2, with a maximum SNP difference of 9,159 and a minimum SNP difference of 6,639 compared to the reference strain YF2203. YF1101, YF1083, YF1078, YF2155, and YF2228 were in clade 3, with a maximum SNP difference of 6,306 and a minimum SNP difference of 6,301 compared to the reference strain YF2203. A threshold of ≤20 SNPs was applied to infer recent clonal transmission, in accordance with accepted standards for *K. pneumoniae* (David et al., 2019).

### Antimicrobial resistance profiles

All 15 *K. pneumoniae* isolates demonstrated universal resistance to carbapenems alongside complete resistance (100%) to cefuroxime, cefoxitin, ceftriaxone, amoxicillin/clavulanic acid, and piperacillin/tazobactam; notably, 14/15 (93.3%) isolates were resistant to ertapenem, imipenem, and cefoperazone/sulbactam, while 12/15 (80.0%) exhibited resistance to ceftazidime and levofloxacin, 11/15 (73.3%) to cefepime, 7/15 (46.7%) to trimethoprim/sulfamethoxazole, 3/15 (20.0%) to tigecycline, and 1/15 (6.7%) to amikacin (Table 2).

**Table 2:** Antimicrobial resistance profiles.

Isolate	Antibiotic (μg/ml)													
	AMC	TZP	CXM	FOX	CAZ	CRO	CSL	FEP	ETP	IPM	AMK	LVX	TGC	SXT
YF1062	≥32 R	≥128 R	≥64 R	≥64 R	≥64 R	≥64 R	≥64 R	≥32 R	≥8 R	≥16 R	≤2 S	≥8 R	≥8 R	≤20 S
YF1072	≥32 R	≥128 R	≥64 R	≥64 R	32 R	≥64 R	≥64 R	≥32 R	≥8 R	≥16 R	≤2 S	4 R	≤0.5 S	≤20 S
YF1073	≥32 R	≥128 R	≥64 R	≥64 R	≥64 R	32 R	16 S	2 S	0.25 S	0.5 S	4 S	≥8 R	2 S	≥320 R
YF1078	≥32 R	≥128 R	≥64 R	≥64 R	32 R	≥64 R	≥64 R	≥32 R	≥8 R	≥16 R	≤2 S	≤0.12 S	≤0.5 S	≤20 S
YF1083	≥32 R	≥128 R	≥64 R	≥64 R	32 R	≥64 R	≥64 R	≥32 R	≥8 R	≥16 R	≤2 S	≤0.12 S	≤0.5 S	≤20 S
YF1101	≥32 R	≥128 R	≥64 R	≥64 R	8 I	32 R	≥64 R	2 S	2 R	≥16 R	≤2 S	≤0.12 S	≤0.5 S	≤20 S
YF2155	≥32 R	≥128 R	≥64 R	≥64 R	≥64 R	≥64 R	≥64 R	≥32 R	≥8 R	8 R	≤2 S	≥8 R	≥8 R	≥320 R
YF2177	≥32 R	≥128 R	≥64 R	≥64 R	≥64 R	≥64 R	≥64 R	≥32 R	≥8 R	≥16 R	≤2 S	≥8 R	1 S	160 R
YF2180	≥32 R	≥128 R	≥64 R	≥64 R	32 R	≥64 R	≥64 R	≥32 R	≥8 R	≥16 R	≤2 S	≥8 R	≥8 R	≥320 R
YF2189	≥32 R	≥128 R	≥64 R	≥64 R	32 R	≥64 R	≥64 R	≥32 R	≥8 R	≥16 R	≤2 S	≥8 R	≤0.5 S	≥320 R
YF2197	≥32 R	≥128 R	≥64 R	≥64 R	8 I	32 R	≥64 R	2 S	≥8 R	≥16 R	≤2 S	4 R	2 S	≤20 S
YF2203	≥32 R	≥128 R	≥64 R	≥64 R	8 I	32 R	≥64 R	2 S	≥8 R	≥16 R	≤2 S	4 R	4 I	≤20 S
YF2227	≥32 R	≥128 R	≥64 R	≥64 R	32 R	≥64 R	≥64 R	≥32 R	≥8 R	4 R	≥64 R	≥8 R	2 S	≥320 R
YF2228	≥32 R	≥128 R	≥64 R	≥64 R	≥64 R	≥64 R	≥64 R	≥32 R	≥8 R	≥16 R	4 S	4 R	4 I	≤20 S
YF2326	≥32 R	≥128 R	≥64 R	≥64 R	≥64 R	≥64 R	≥64 R	≥32 R	2 R	≥16 R	≤2 S	≥8 R	1 S	≥320 R

AMC, amoxicillin/clavulanic acid; TZP, piperacillin-tazobactam; CXM, cefuroxime; FOX, cefoxitin; CAZ, ceftazidime; CRO, ceftriaxone; CSL, cefoperazone/sulbactam; FEP, cefepime; ETP, ertapenem; IPM, imipenem; AMK, amikacin; LVX, levofloxacin; TGC, tigecycline; SXT, trimethoprim/sulfamethoxazole.

### Molecular characteristics of resistance and virulence genes and plasmids

**Figure 2:**
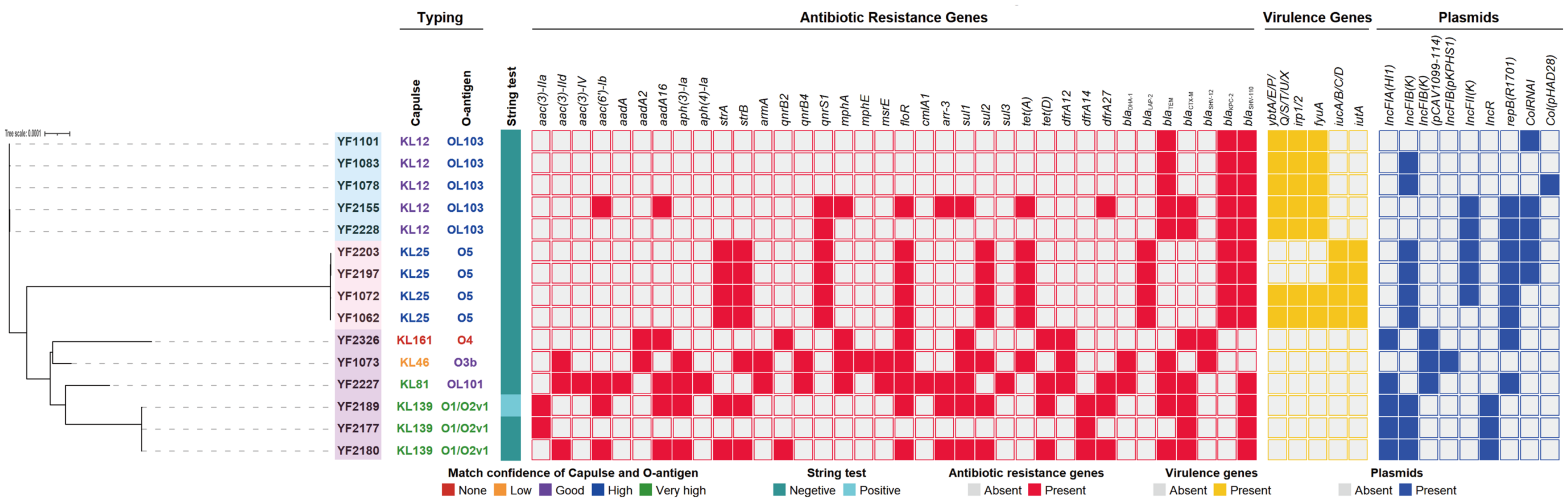
Phylogenetic analysis and characteristics of *K. pneumoniae* complex phylogroups, capsular serotype, LPS O-antigen, string test, antibiotic resistance genes, virulence genes, and plasmid replicon types. The KL/O match confidence and string test of *K. pneumoniae* complex are differentiated by color. Antibiotic resistance genes, virulence genes, and plasmid replicon types are denoted by filled squares for the presence and empty squares for absence.

As shown in Figure 2, all 15 isolates of *K. pneumoniae* belong to ST37, with most strains in this study producing both ESBLs and carbapenemase simultaneously. Among the 15 CRKP strains, *bla*_KPC-2_ was detected in 9 strains (60.0%). The positive rates of the *bla*_SHV-110_, *bla*_SHV-12_, *bla*_TEM-1D_ and *bla*_TEM-215_ genes were 13/15 (86.7%), 2/15 (13.3%), 8/15 (53.3%) and 1/15 (6.7%) respectively. The *bla*_CTX-M-27_, *bla*_CTX-M-15_, and *bla*_CTX-M-3_ genes were, respectively, found in the strains of 4/15 (26.7%), 2/15 (13.3%) and 2/15 (13.3%). For *bla*_LAP-2_ (4/15, 26.7%) and *bla*_DHA-1_ (2/15, 13.3%), the levels of the observed resistance genes were relatively low. Other β-lactamase genes, such as *bla*_OXA_, *bla*_ACT_, *bla*_GES_, *bla*_SME_, *bla*_NDM_, *bla*_IMP_, and *bla*_VIM_, were not detected in any isolates. In addition, other resistance genes were identified, including the aminoglycoside resistance genes *aac(3)-IIa*, *aac(3)-IId*, *aac(3)-IV*, *aac(6′)-Ib*, *aadA*, *aadA2*, *aadA16*, *aph(3)-Ia*, *aph(4)-Ia*, *strA*, *strB*, *armA*; the fluoroquinolone resistance genes *qnrS1*, *qnrB2*, *qnrB4*; the macrolide resistance genes *mphA*, *mphE*, *msrE*; the phenicol resistance genes *floR*, *cmlA1*; the rifampicin resistance gene *arr-3*; the sulfamethoxazole resistance genes *sul1*, *sul2*, *sul3*; the tetracycline resistance genes *tet(A)*, *tet(D)* and the trimethoprim resistance genes *dfrA12*, *dfrA14*, *dfrA27*.

To verify the virulence profile of the CRKP strain, we characterized 38 virulence-associated genes. *K. pneumoniae* strains YF1062, YF1072, YF2197 and YF2203 all harbored aerobactin (*iucA/B/C/D*) and aerobactin receptor (*iutA*). Besides, yersiniabactin (*ybtA/E/P/Q/S/T/U/X*), yersiniabactin biosynthesis (*irp1/2*) and yersiniabactin receptor (*fyuA*) were identified in strains YF1062, YF1072, YF1078, YF1083, YF1101, YF2155, and YF2228. All these strains carried the yersiniabactin gene *ybt9* or *ybt15* on ICEKp3. Serotype analysis of WGS data revealed the presence of five types of *wzi* alleles (12, 81, 141, 150, and 372) and six types of O-loci (O5, OL103, OL101, O1/O2v1, O3b, and O4). The isolates covered 6 K-loci, showing a diverse distribution. KL12 was the most common (5/15, 33.3%), followed by KL25, KL139, KL81, KL46, and one *K. pneumoniae* strain with KL161, but the matching confidence was none. The proportion of OL103 (5/15, 33.3%) is the highest, followed by O5, O1/O2v1, OL101, O3b and O4 (match confidence: 0%). Four of the CRKP that indicated strong virulence had a KL25 capsular serotype and O5 LPS serotype.

Plasmid replicon typing of the 15 *K. pneumoniae* isolates revealed a diverse array of plasmid types, with all strains harboring at least one plasmid. The most prevalent replicon types were IncFIB (K) (12/15, 80.0%), either in its classic form or as an identified variant, and repB (R1701) (8/15, 53.3%). The primary characteristic of the nine *bla*_KPC-2_ positive isolates was the presence of the IncFII(K)/repB(R1701) plasmid, while the remaining KPC-2 may be integrated within the chromosome. A concerning convergence was observed in 7 strains (46.7%), which simultaneously carried the virulence plasmid IncFIB (K) and resistance plasmids (IncFII (K) or IncFIA (HI1)). Among these, four strains (YF1062, YF1072, YF2197, and YF2203) were identified as belonging to the *iuc3* lineage, located on IncFIB (K) plasmid. The identical and complex plasmid profiles in sequentially recovered isolates (YF2197 and YF2203) from a single patient underscores the stability and transmissibility of these converged plasmids.

### Pathogenic analysis shows the emergence of hypervirulent ST37 CRKP

**Figure 3:**
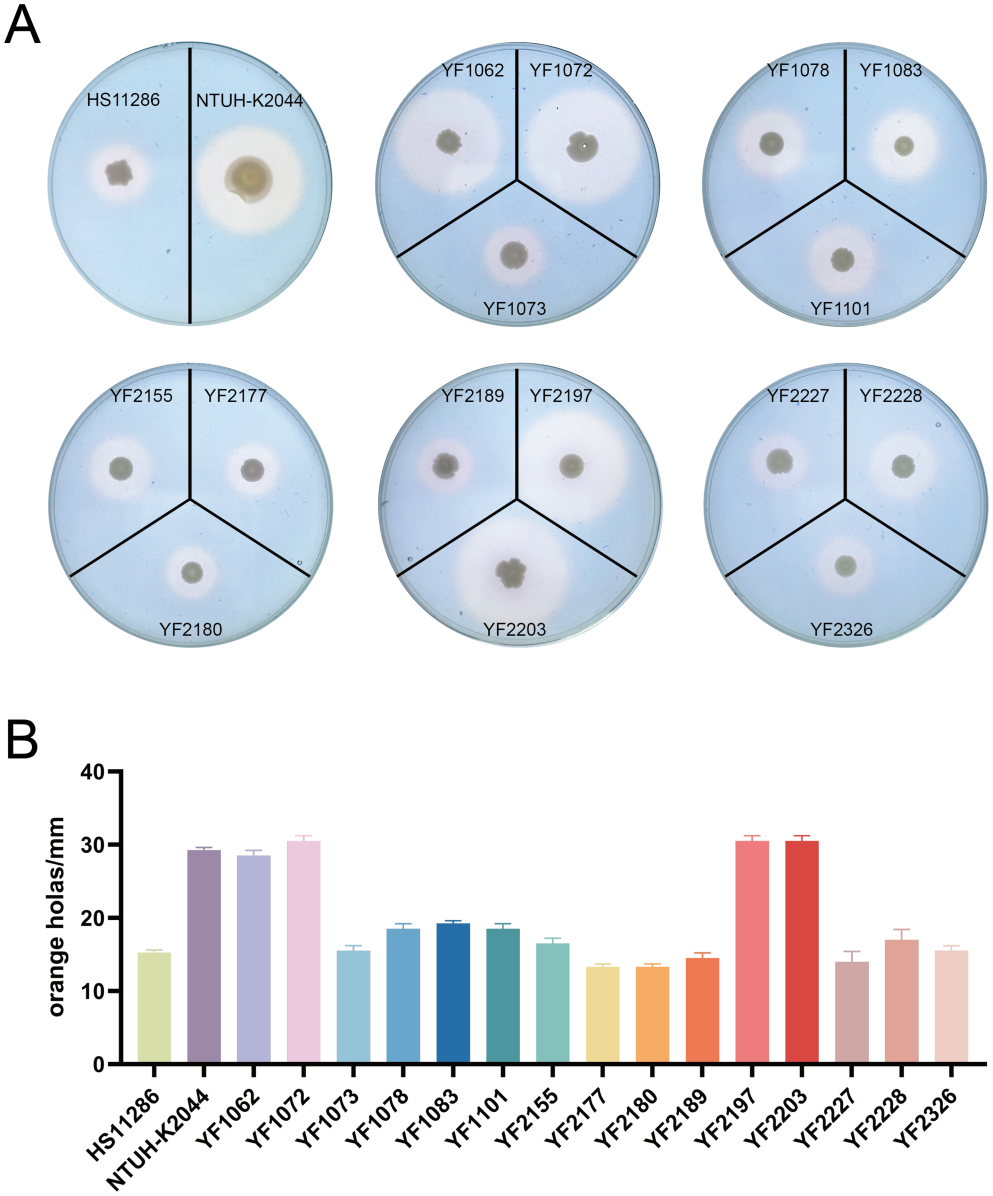
Siderophore production in *K. pneumoniae*. **(A)** Representative image of *K. pneumoniae* forming orange halos on CAS agar plates. **(B)** Diameter of the orange halos.

**Figure 4:**
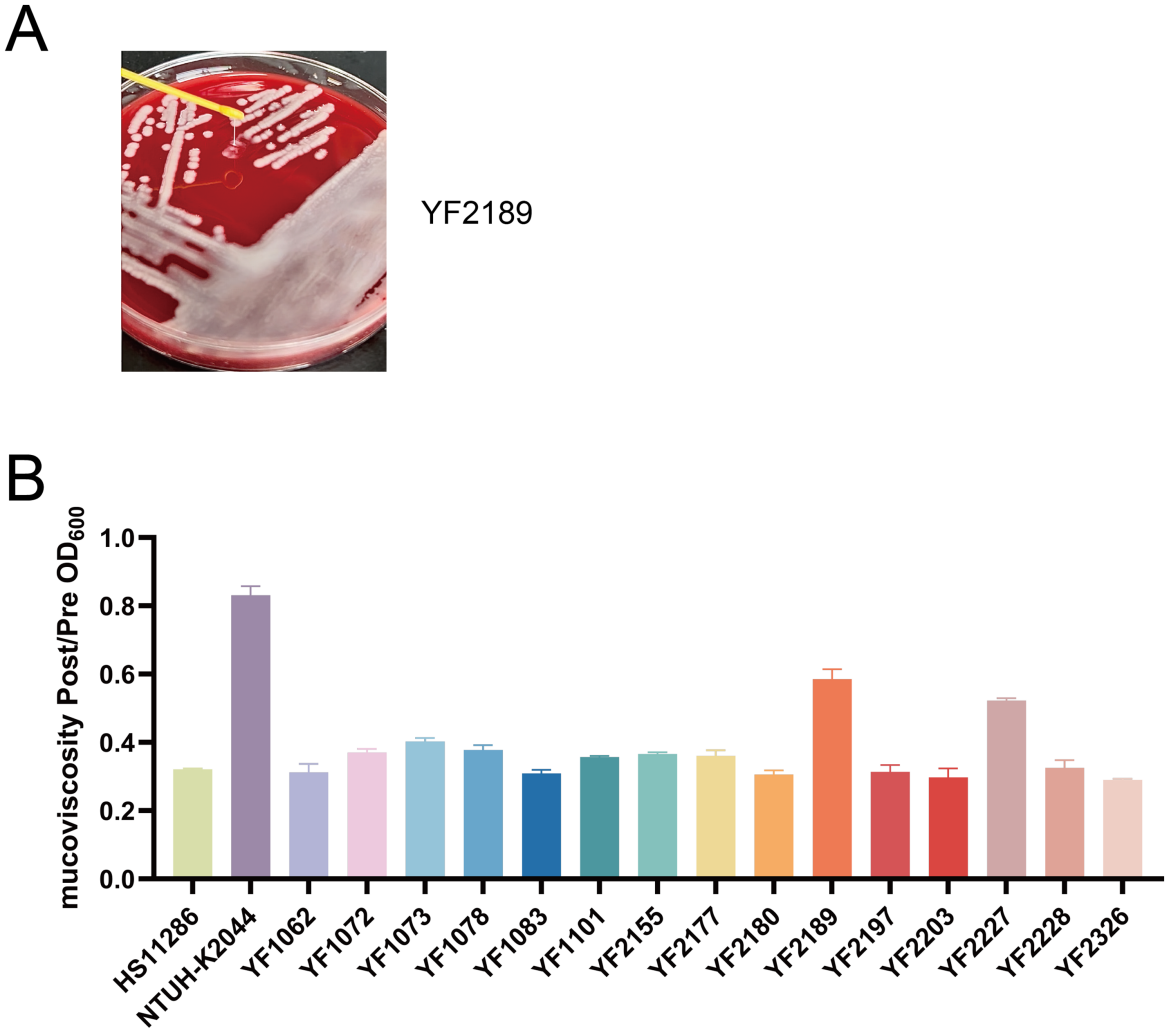
Mucoviscosity of *K. pneumoniae*. **(A)** String test. **(B)** Semi-quantitative mucoviscosity assessment.

**Figure 5:**
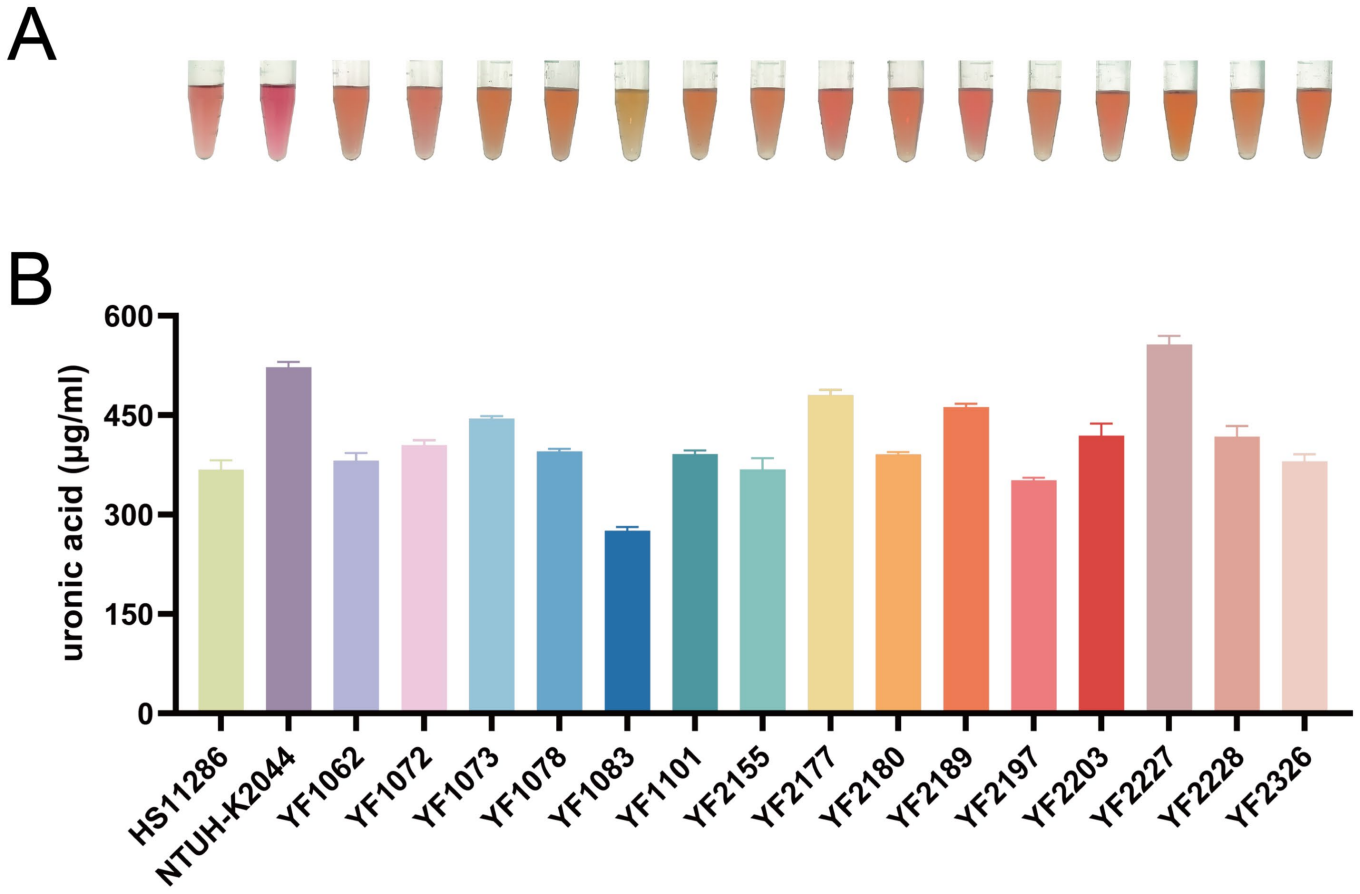
Capsular polysaccharide production in *K. pneumoniae*. **(A)** Representative image showing pink coloration indicative of capsular polysaccharide detection. **(B)** Levels of uronic acid.

**Figure 6:**
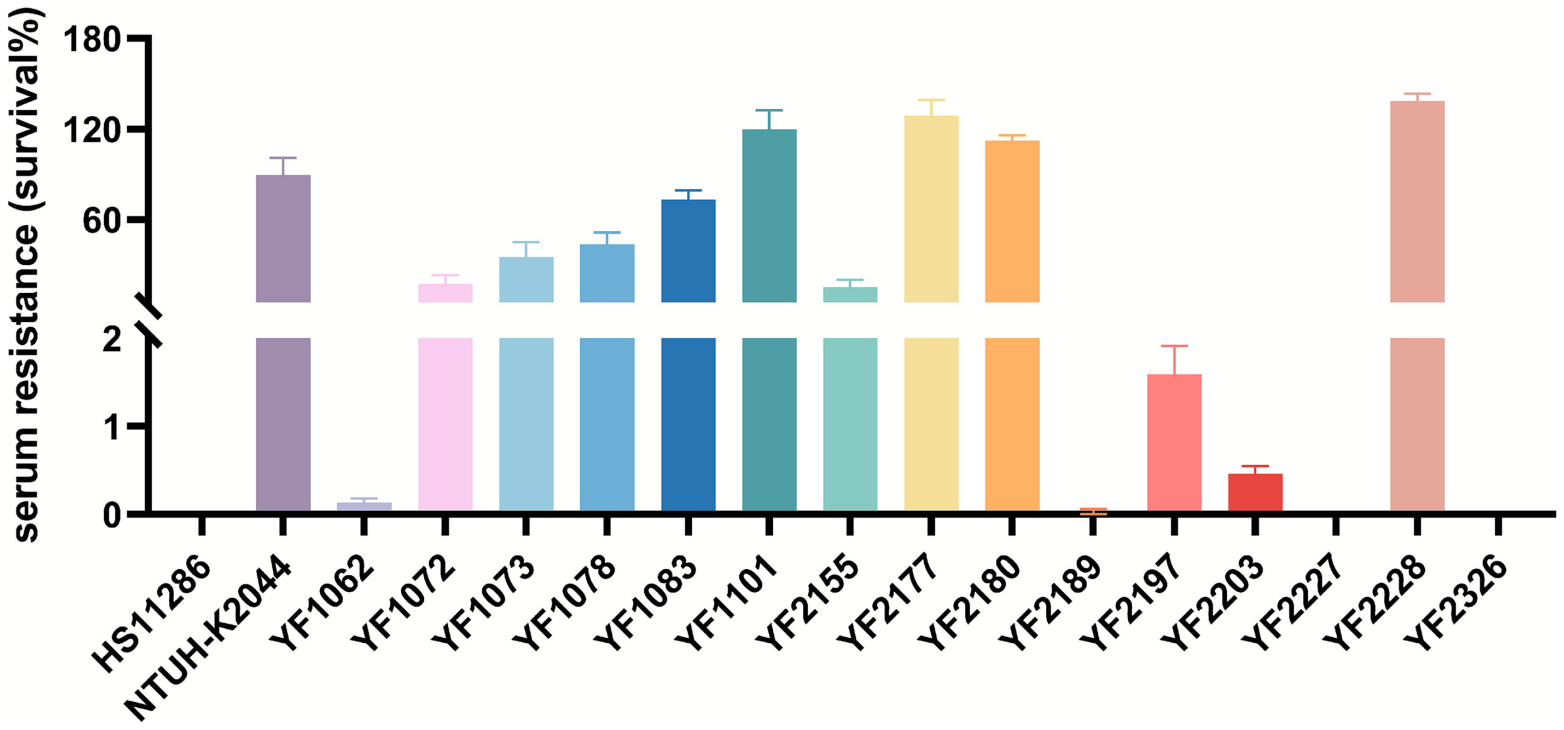
Serum resistance of *K. pneumoniae*. The survival rate is defined as the proportion of viable bacteria remaining following a two-hour incubation with the serum.

The 15 strains of ST37 CRKP analyzed in this study were evaluated using various methods to detect virulence factors. Quantitative siderophore assays revealed that strains YF1062, YF1072, YF2197, and YF2203 produced siderophore levels comparable to the positive control NTUH-K2044 and significantly higher than the negative control HS11286 (Figure 3). Strains harboring the aerobactin genes (*iuc*, *iutA*) exhibited high siderophore production, whereas those carrying only the yersiniabactin genes (*ybt*, *irp*, *fyuA*) showed an intermediate level. In contrast, strains lacking both gene clusters produced minimal siderophore, at a level comparable to that of the reference strain HS11286. These results revealed that the *iuc3* gene cluster greatly contributes to iron acquisition. String test results indicated that no isolates except YF2189 exhibited a hypermucoviscous phenotype (Figure 4A). Moreover, semi-quantitative mucoviscosity measurements corroborated these findings, with YF2189 demonstrating significantly higher mucoviscosity (Figure 4B). In terms of capsular polysaccharide production, measured via uronic acid levels, results indicated that YF2227 produced uronic acid levels equivalent to NTUH-K2044, followed by YF2177, YF2189, and YF1073. The uronic acid production in the remaining strains was similar to that of the negative control HS11286, while YF1083 manifested the lowest levels (Figure 5). Serum resistance assays demonstrated that YF1101, YF2177, YF2180, and YF2228 exhibited serum resistance similar to NTUH-K2044 (Figure 6).

**Figure 7:**
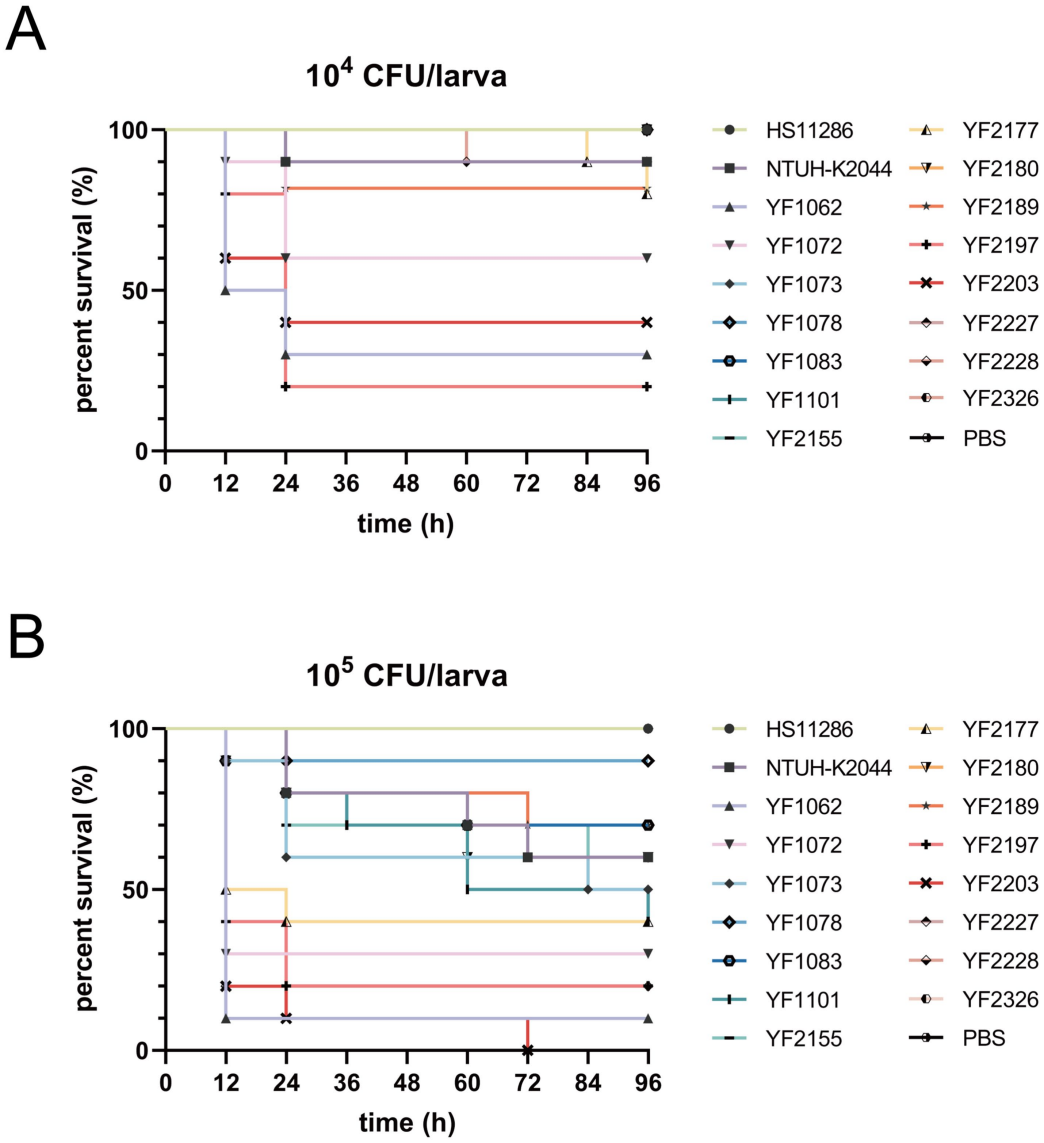
Survival curves generated by the *G. mellonella* infection model. Ten larvae per group were injected with 10 μL of each *K. pneumoniae* isolates at **(A)** 10^6^ CFU/mL and **(B)** 10^7^ CFU/mL.

Furthermore, virulence was assessed using the *G. mellonella* infection model. At 4 days post-infection, strains YF1062, YF1072, YF2197, and YF2203 displayed high virulence, resulting in significantly reduced larval survival rates, with consistent results observed for inocula of 10^4^ and 10^5^ CFU (Figure 7). Virulence potential among CRKP strains exhibited substantial heterogeneity in *G. mellonella* larvae infected with 10^5^ CFU/larva. Strains YF1062 and YF2203 was significantly more virulent than other isolates, with YF1062 reducing larval survival to ≤ 10% within 12 h and YF2203 achieving 0% survival by 72 h. Strains YF1072 and YF2197 displayed high virulence, yielding survival rates of ≤30% at 24 h. Intermediate virulence was observed in YF2228, YF2177, YF1101, YF1073, YF2155, and YF2180, exhibiting survival rates between 40 and 60% at 96 h. Strains YF1083, YF2189, YF1078, YF2227, and YF2326 showed the lowest virulence, with survival rates exceeding 70% at 96 h. Thus, these four aerobactin-positive strains with high *G. mellonella* lethality were designated hvKP. Collectively, these findings demonstrated that ST37 CRKP, in addition to its hallmark multidrug resistance, harbors hypervirulent isolates, posing a significant clinical threat and underscoring the urgent need for enhanced surveillance and therapeutic strategies.

## Discussion

The global spread of carbapenem-resistant *K. pneumoniae* (CRKP) poses a critical threat to public health, particularly in healthcare settings where these pathogens contribute to high mortality rates due to limited treatment options (Shah et al., 2025). While MDR *K. pneumoniae* has long been a concern, the emergence of hypervirulent CRKP (hv-CRKP) strains, combining extensive antibiotic resistance with enhanced pathogenicity, represents an alarming evolutionary convergence (Zhu et al., 2024; Martin and Bachman, 2018; Kochan et al., 2023). Historically, hvKP was associated with community-acquired infections and susceptible to most antibiotics, whereas CRKP strains were primarily nosocomial pathogens with high resistance but relatively lower virulence (Al Ismail et al., 2025; Chen et al., 2024b). Sequence type 37 (ST37), a high-risk CRKP clone increasingly reported in Asia, has been primarily linked to AMR rather than hypervirulence (Zhu et al., 2016; Xia et al., 2017; Taniguchi et al., 2017; Krishnan et al., 2025). This comprehensive phylogenetic analysis of 1,251 global ST37 *K. pneumoniae* isolates provided a detailed map of the population structure and dissemination of this clone. A central finding of our study is the identification and characterization of 15 clinical isolates that cluster within the predominant Clades 1, 2, and 3. This placement indicates that our locally collected strains are not sporadic outliers but belong to the globally circulating and evolutionarily successful sub-lineages of ST37. Here, we characterize a collection of ST37 CRKP isolates, revealing a subset that exhibits both extreme drug resistance and hypervirulent traits, signaling a dangerous shift in the epidemiology of this pathogen.

The universal resistance to carbapenems and near-universal resistance to expanded-spectrum cephalosporins and β-lactam/β-lactamase inhibitor combinations observed in all 15 ST37 *K. pneumoniae* isolates, underscores a critical depletion in therapeutic options for infections caused by these strains. This resistance phenotype is mechanistically anchored in a complex repertoire of β-lactamase genes, dominated by the carbapenemase gene *bla*_KPC-2_ (60.0%) alongside prevalent ESBL genes [*bla*_SHV-110_ (86.7%), *bla*_TEM-1D_ (53.3%), and various *bla*_CTX-M_ alleles]. The absence of metallo-β-lactamases (MBLs) like NDM, IMP, or VIM is noteworthy, suggesting a KPC-driven resistance landscape distinct from regions where MBLs dominate CRKP epidemiology. This genotypic profile, coupled with a broad array of resistance genes against aminoglycosides (*aac*, *aad*, *aph* families), fluoroquinolones (*qnrS1*, *qnrB* variants), sulfonamides (*sul*), trimethoprim (*dfrA*), and tetracyclines (*tet*), paints a picture of near-pan-drug resistance, leaving only aminoglycosides (amikacin resistance at 6.7%) and tigecycline (resistance at 20.0%) as potentially viable, albeit fragile, last-resort options.

The pivotal finding is the identification of hypervirulent phenotypes within this MDR lineage. Four isolates with a highly consistent genetic relationship (YF1062, YF1072, YF2197, YF2203) exhibited definitive hypervirulence markers (Huynh et al., 2020; Thirugnanasambantham et al., 2025): (i) carriage of the complete aerobactin system (*iucABCD*/*iutA*), a key virulence determinant strongly associated with invasive disease and worse clinical outcomes; (ii) significantly elevated siderophore production comparable to the hypervirulent control strain NTUH-K2044; and (iii) profound lethality in the *G. mellonella* infection model, causing rapid larval death even at moderate inocula (10^4^ CFU/larva). This association between the *iucABCD*/*iutA* locus and hypervirulence in the *G. mellonella* infection model is consistent with established literature, confirming the functional impact of this siderophore system on pathogenicity. The isolates YF1062 and YF1072 were both obtained from patients in the ICU, with both isolates having closely aligned collection dates. Two other hv-CRKP isolates YF2197 and YF2203 were collected from a patient with cerebral infarction in the neurology ward. They were isolated from sputum and stool specimens, respectively, with a three-day interval between collections. Crucially, these hypervirulent strains retained the MDR profile characteristic of the ST37 cohort, including carbapenem resistance primarily mediated by KPC-2. This represents a significant evolution in the ST37 lineage, historically recognized for its multidrug resistance but not typically associated with classic hypervirulence.

The genomic diversity observed, particularly in K- and O-loci (6 types, KL12/OL103 dominant at 33.3%), highlights heterogeneity within this ST37 cluster. However, a critical correlation emerged: all four hv-CRKP strains possessed the KL25 capsule serotype and O5 LPS serotype. This specific combination (KL25/O5) warrants further investigation as a potential predictor or facilitator of the hypervirulent phenotype within ST37 CRKP, reminiscent of the association of KL1/KL2 with classical hvKP lineages like ST23. While other virulence-associated systems like yersiniabactin (*ybt*, *irp*, *fyuA* on ICEKp3) were detected in several isolates (including some non-hv strains), their presence alone was insufficient to confer the hypervirulent phenotype observed in the aerobactin-positive isolates. This reinforces the primary role of aerobactin in driving hypervirulence in this context. Hypermucoviscosity serves as a significant *in vitro* parameter for identifying hvKP and has traditionally defined this pathotype (Liu et al., 2014; Zhang et al., 2016). While most hypermucoviscous *K. pneumoniae* strains harbor *rmpA* or *rmpA2*, exceptions exist (Altayb et al., 2022). Although isolates lacked these common regulators (*rmpADC*/*rmpA2*), they harbored other regulators (*rcsA* and *rcsB*), indicating an alternative mechanism for hypermucoviscosity. However, the association between the hypermucoviscous phenotype and virulence remains contentious (Lin et al., 2011; Hetta et al., 2025). Other virulence assays revealed additional heterogeneity: a single isolate (YF2189) exhibited hypermucoviscosity, one isolate (YF2227) showed high capsular polysaccharide production, and several demonstrated serum resistance. These traits often associated with hvKP but not uniformly present in our hv-CRKP subset, indicating nuanced variations in virulence factor expression within the ST37 background.

The convergence of resistance and hypervirulence in ST37 CRKP carries profound clinical implications. ST37 is an increasingly reported high-risk clone globally, particularly in Asia, known for its propensity to acquire resistance determinants. Its evolution to incorporate hypervirulence mechanisms like aerobactin significantly elevates its threat level. Infections caused by such convergent strains, combining resistance to last-line antibiotics like carbapenems with enhanced invasive potential, pose an almost insurmountable challenge to clinical management and are likely associated with high morbidity and mortality. The co-occurrence of *bla*_KPC-2_ and *iuc3* on mobile genetic elements within the same strain is a particularly concerning scenario, potentially enabling the simultaneous dissemination of both traits. Our finding that only 4 of the 15 ST37 isolates were hypervirulent, despite all sharing the same ST and core resistance profile, suggests that the acquisition of key virulence loci like the aerobactin cluster is a critical, possibly recent, evolutionary step within this lineage. However, the generalizability of these results should be interpreted with caution due to the limited sample size (*n* = 15). Population-based surveillance studies are needed to elucidate the epidemiological significance of hypervirulent ST37 CRKP. Moreover, the specific genetic context (plasmids, integrative conjugative elements) carrying the *iuc3* and *bla*_KPC-2_ warrants detailed investigation to understand transmission dynamics. *In vitro* virulence assays, while informative, require correlation with *in vivo* mammalian models and clinical outcome data to fully assess the pathogenic potential.

In conclusion, we report the emergence of hypervirulent CRKP within the ST37 lineage, characterized by the carriage of the aerobactin system (*iucABCD*/*iutA*), high siderophore production, and exceptional lethality in an infection model, all coexisting with a formidable array of AMR genes, most notably *bla*_KPC-2_. The association of this hypervirulent phenotype with the KL25/O5 serotype combination provides a potential marker for surveillance. The emergence of ST37 CRKP strains co-harboring the globally prevalent KPC-2 carbapenemase and aerobactin-mediated hypervirulence represents a serious epidemiological threat. This convergence threatens to erode the already limited therapeutic options for severe *K. pneumoniae* infections and highlights the urgent need for enhanced genomic surveillance targeting virulence genes in CRKP populations, alongside accelerated development of novel antimicrobials and therapeutic strategies targeting both resistance and virulence pathways. The identification of ST37 as a platform for this convergence necessitates focused infection control and research efforts to mitigate its potential spread and impact.

## Data Availability

The datasets presented in this study can be found in online repositories. The names of the repository/repositories and accession number(s) can be found in the article/Supplementary material.

## Footnotes

1
https://github.com/tseemann/mlst

2
https://genepi.food.dtu.dk/resfinder

3
https://github.com/katholt/Kleborate/

4
https://cge.food.dtu.dk/services/PlasmidFinder/
